# Association between the alanine aminotransferase/aspartate aminotransferase ratio and new-onset non-alcoholic fatty liver disease in a nonobese Chinese population: a population-based longitudinal study

**DOI:** 10.1186/s12944-020-01419-z

**Published:** 2020-11-25

**Authors:** Yang Zou, Ling Zhong, Chong Hu, Guotai Sheng

**Affiliations:** 1 Cardiology Department, Jiangxi Provincial People’s Hospital Affiliated to Nanchang University, Nanchang, Jiangxi Province China; 2 Medical Department of Graduate School, Nanchang University, Nanchang, Jiangxi Province China; 3 Pediatrics Department, Lishui People’s Hospital, Lishui, Zhejiang Province China; 4 Gastroenterology Department, Jiangxi Provincial People’s Hospital Affiliated to Nanchang University, Nanchang, Jiangxi Province China

**Keywords:** Longitudinal study, Non-alcoholic fatty liver disease, Risk factor, Nonobese, ALT/AST ratio

## Abstract

**Background:**

The alanine aminotransferase (ALT)/aspartate aminotransferase (AST) ratio has been considered an alternative marker for hepatic steatosis. However, few studies have investigated the association of the ALT/AST ratio with non-alcoholic fatty liver disease (NAFLD) in nonobese people.

**Methods:**

A total of 12,127 nonobese participants who were free of NAFLD participated in this study. The participants were divided into quintiles of the ALT/AST ratio. Multiple Cox regression models were used to explore the association of the ALT/AST ratio with new-onset NAFLD.

**Results:**

During the five-year follow-up period, 2147 individuals (17.7%) developed new-onset NAFLD.

After adjusting for all non-collinear covariates, the multiple Cox regression analysis results showed that a higher ALT/AST ratio was independently associated with new-onset NAFLD in nonobese Chinese (adjusted hazard ratios [aHRs]: 2.10, 95% confidence intervals: 1.88, 2.36). The aHRs for NAFLD across increasing quintiles of the ALT/AST ratio were 1, 1.63 (1.30, 2.04), 2.07 (1.65, 2.60), 2.84 (2.33, 3.48) and 3.49 (2.78, 4.39) (*P* for trend< 0.001). The positive association was more significant among people with high blood pressure, high blood lipids and hyperglycaemia, as well as in men. Additionally, the regression spline showed that the saturation effect of the ALT/AST ratio on NAFLD risk was at 0.93 in this study population, which was 1.22 in males and 0.89 in females.

**Conclusions:**

In nonobese Chinese individuals without NAFLD at baseline, the increase in the ALT/AST ratio is closely associated with the risk of new-onset NAFLD.

**Supplementary Information:**

The online version contains supplementary material available at 10.1186/s12944-020-01419-z.

## Background

Non-alcoholic fatty liver disease (NAFLD) has become an increasingly serious public health problem worldwide. About 1/4 (22.10–28.65%) of the world’s population suffers from NAFLD [1–3]. Compared with common chronic diseases such as hypertension (30.0–32.2%) and diabetes (22.9–37.5%) [4, 5], the epidemic scale of NAFLD is equally shocking and will continue to increase [6]. The impact of NAFLD is systemic. It is not only related to the development and deterioration of liver diseases but also to cardiovascular disease, metabolic disease, malignant tumours and other diseases [1, 3, 7–9]. Currently, NAFLD is the second leading indication for liver transplantation in the USA [10, 11]. NAFLD and its complications have brought an enormous burden to the health care system [6, 9–12].

Clinically, obesity is closely associated with NAFLD [1, 3, 13], but it is worth noting that in the general population, there are still many people with a normal body mass index (BMI) who are diagnosed with NAFLD. In the third National Health and Nutrition Inspection Survey of America, it was reported that liver steatosis could be detected by ultrasound in 7.4% of nonobese adults [14], and in Asia, the figure was as high as 8–19% [15]. The difference in NAFLD prevalence between the Asian population and the European and American populations suggests that there may be other more important metabolic risk factors in these populations with normal BMI, and identifying these risk factors may help people of normal BMI to prevent NAFLD as early as possible. Additionally, early detection of nonobese NAFLD can reduce the risk of cardiovascular disease and diabetes [16–18].

Aspartate aminotransferase (AST) and alanine aminotransferase (ALT) are often used to indicate the quality of liver function. In previous studies, ALT has been shown to be associated with NAFLD. Even ALT values within the normal reference range have been associated with a risk of NAFLD [2, 19]. The ALT/AST ratio can be used to evaluate the degree of hepatic fat infiltration and hepatic steatosis [20, 21]. In a recent Framingham study, it was shown that the ALT/AST ratio could identify hepatic steatosis more accurately than using ALT or AST alone [21]. Presently, there are limited studies that have reported on the association of the ALT/AST ratio with NAFLD risk. Only a few cross-sectional studies have found a positive correlation of NAFLD risk with the ALT/AST ratio [22–25]. Nevertheless, the association of the ALT/AST ratio with NAFLD in nonobese individuals remains inconclusive. The present study was designed to elucidate the association of the ALT/AST ratio with new-onset NAFLD in nonobese Chinese people.

## Methods

### Study population and design

This study was a secondary analysis of a longitudinal cohort study of 16,173 health examination participants recruited in Wenzhou People’s Hospital from January 2010 to December 2014. The research data came from https://datadryad.org (10.5061/dryad.1n6c4) [26]. The database contains the following variables: sex, age, height, weight, alanine aminotransferase (ALT), systolic blood pressure (SBP), fasting plasma glucose (FPG), diastolic blood pressure (DBP), high-density lipoprotein-cholesterol (HDL-C), blood urea nitrogen (BUN), aspartate aminotransferase (AST), globulin (GLB), uric acid (UA), triglyceride (TG), creatinine (Cr), direct bilirubin (DBIL), low-density lipoprotein-cholesterol (LDL-C), gamma-glutamyl transferase (GGT), albumin (Alb), total protein (TP), total cholesterol (TC), alkaline phosphatase (ALP), and total bilirubin (TB). Due to the secondary data analysis nature of this research, and since the Ethics Committee of Wenzhou People’s Hospital has approved the previous study [26], this study did not need to apply for ethical approval again. In this longitudinal observational cohort study, patients who included the following criteria at baseline were excluded: (i) excessive drinking (female: > seventy g/w, male: >one hundred forty g/w); (ii) any liver disease at baseline; (iii) taking medication at baseline; (iv) obese (BMI ≥25 kg/m^2^); (v) LDL-C above the normal reference range (> 3.12 mmol/L); and (vi) incomplete baseline ALT or AST data.

### Data acquisition

As mentioned earlier [26], all participants were examined at baseline by trained medical personnel, and basic clinical data such as age, sex, height, weight, DBP and SBP were recorded using a standardized self-filling spreadsheet. Venous blood was extracted after a night of fasting and analysed using an automatic biochemical analyser (Abbott AxSYM), which was operated by trained medical personnel, and haematological parameters were collected.

### Diagnosis and follow-up of NAFLD

Abdominal colour ultrasound was performed and evaluated by trained professional and technical personnel. Of the five known criteria, the diagnosis of NAFLD must include diffuse hyperechoic of the liver and any of the following four items: (i) mild to moderately enlarged liver with blunt, rounded edges; (ii) unclear or nonintact display of envelope of right liver lobe and diaphragm; (iii) weakened hepatic blood flow signal with normal blood flow distribution; and (iv) reduced visibility of detailed structure in the liver [27]. This study population was prospectively followed for 5 years with evaluations once a year; the primary endpoint was new-onset NAFLD.

### Statistical analysis

The statistical analysis of this study mainly included the following three steps.

- Step 1 (Handling missing data): The dataset contained 11,922 missing values (Supplementary Table 1, Additional file 1), accounting for 3.93% of the total data. To maximize statistical power, the researchers used multiple multivariate imputations [28]. As a sensitivity analysis, they also compared the complete data after multiple imputation with the original data (Additional file 1: Supplementary Table 2, Additional file 1, *P* > 0.05). In this study, all multiple analyses were carried out in each imputed dataset and original data separately, with the results pooled based on Rubin’s rules [29].
- Step 2 (Statistical description and simple statistical analysis): (i) continuous variable: normality was examined by the Kolmogorov-Smirnov test and QQ plots. When a variable conformed to a normal distribution, it was presented as the mean ± standard deviation; otherwise, it was described by the median (interquartile range); statistical comparisons between the groups were conducted using nonparametric Kruskal–Wallis H tests or one-way ANOVA; and (ii) qualitative variables were presented as frequencies (%), and the differences between the groups were evaluated by the weighted χ2-test.
- Step 3 (Correlation analysis): First, the collinearity of the variables was examined by calculating the variance inflation factor using multiple linear regression analysis [30]; the details of the collinearity diagnostics steps are shown in Supplementary Table 3 (Additional file 1). Second, multiple Cox regression models were used to calculate the hazard ratios (HRs) and 95% confidence intervals (CI) of the association between ALT/AST ratio and NAFLD risk, and possible special populations were examined in a predesignated subgroup analyses (stratification by age, sex, SBP, DBP, GGT, FPG, TC and TG according to the clinical cut-off points). The adjustment of variables in this study shows different degrees of adjustment results based on the statement of Strengthening the Reporting of Observational Studies in Epidemiology (STROBE) [31]. The crude model was an unadjusted model, and model I only adjusted for the basic demographic data (sex and age). Model II adjusted for the variable that had a > 10% impact on the association between the ALT/AST and NAFLD risk (sex, age, GGT, DBIL, TG, HDL-C and BMI) [32]. Model III adjusted for the variables with *P* < 0.1 in the simple Cox regression models + Model II. Model IV adjusted for all non-collinear variables. Finally, the generalized additive model (GAM, with cubic spline smoothing) was used to simulate the association between the ALT/AST ratio as a continuous variable and NAFLD risk [33], the recursive algorithm was used to fit the model with the maximum likelihood, and the inflection point of the nonlinear correlation was calculated.

The Empower (R, version 2.20) and R (version 3.4.3) statistical packages were used for the analyses. *P* < 0.05 (2-tailed) was the significance threshold.

## Results

This longitudinal cohort study included 12,127 participants (6646 men and 5481 women) who met the inclusion and exclusion criteria, with an average BMI of 21.61 ± 2.04 kg/m^2^ and an average age of 43.28 ± 14.95 years. Table 1 lists the basic characteristics of the participants based on the quintiles of the ALT/AST ratio. People with a high ALT/AST ratio had higher weight, ALP, height, DBP, SBP, BMI, GGT, LDL-C, ALT, TP, AST, TG, ALB, TC, TB, UA, Cr and FPG, while age, GLB, DBIL, BUN and HDL-C were lower. During the five-year follow-up period, 2147 individuals (17.7%) developed new-onset NAFLD, and the incidence of NAFLD increased gradually by increasing quintile of the ALT/AST ratio (Q1: 5.7% vs Q2: 9.6% vs Q3: 13.9% vs Q4: 23.09% vs Q5: 35.78%).

**Table 1:** Baseline Characteristics of participants

ALT/AST ratio	Quintile 1 (< 0.6)	Quintile 2 (≥0.6, < 0.71)	Quintile 3 (≥0.71, < 0.84)	Quintile 4 (≥0.84, < 1.04)	Quintile 5 (≥1.04)	*P*-value
N (%)	2387 (19.68%)	2428 (20.02%)	2374 (19.58%)	2512 (20.71%)	2426 (20.01%)	
Clinical data						
Age, years	41.00 (32.00–54.00)	40.00 (32.00–51.00)	41.00 (32.00–53.00)	40.00 (31.00–51.00)	39.00 (31.00–50.00)	< 0.001
Sex						< 0.001
Female	1123 (47.05%)	1153 (47.49%)	1104 (46.50%)	1076 (42.83%)	1025 (42.25%)	
Male	1264 (52.95%)	1275 (52.51%)	1270 (53.50%)	1436 (57.17%)	1401 (57.75%)	
NAFLD	136 (5.70%)	233 (9.60%)	330 (13.90%)	580 (23.09%)	868 (35.78%)	< 0.001
Weight, kg	56.22 ± 8.06	57.59 ± 8.03	59.23 ± 8.05	61.91 ± 8.04	64.48 ± 7.82	< 0.001
Height, m	1.64 ± 0.08	1.65 ± 0.08	1.66 ± 0.08	1.67 ± 0.07	1.69 ± 0.07	< 0.001
BMI, kg/m^2^	20.80 (19.31–22.26)	21.26 (19.70–22.78)	21.71 (20.08–23.05)	22.22 (20.67–23.58)	22.80 (21.46–23.91)	< 0.001
SBP, mmHg	122.63 ± 19.05	121.17 ± 17.92	121.69 ± 16.76	122.94 ± 15.67	123.57 ± 14.94	< 0.001
DBP, mmHg	72.00 (65.00–80.00)	71.00 (65.00–79.00)	73.00 (66.00–80.00)	74.00 (67.00–81.00)	74.00 (68.00–82.00)	< 0.001
Laboratory data						
ALP, U/L	68.70 ± 24.64	69.12 ± 20.28	70.94 ± 22.24	74.32 ± 23.29	78.52 ± 23.98	< 0.001
GGT, U/L	18.00 (14.00–23.00)	19.00 (15.00–24.00)	21.00 (17.00–28.00)	24.00 (19.00–34.00)	32.00 (23.00–51.00)	< 0.001
ALT, U/L	10.00 (9.00–12.00)	13.00 (11.00–15.00)	16.00 (14.00–19.00)	20.00 (17.00–24.00)	31.00 (24.00–41.00)	< 0.001
AST, U/L	20.00 (18.00–24.00)	20.00 (17.00–23.00)	21.00 (18.00–24.00)	22.00 (19.00–25.00)	24.00 (21.00–30.00)	< 0.001
TP, g/L	73.86 ± 4.40	73.74 ± 4.11	73.61 ± 4.17	73.90 ± 4.23	74.16 ± 4.34	< 0.001
ALB, g/L	44.18 ± 2.80	44.29 ± 2.79	44.42 ± 2.61	44.76 ± 2.81	45.05 ± 2.74	< 0.001
GLB, g/L	29.68 ± 4.15	29.45 ± 3.94	29.19 ± 3.81	29.14 ± 3.82	29.11 ± 4.13	< 0.001
TB, μmol/L	11.00 (8.70–14.40)	11.30 (8.60–14.20)	11.20 (8.90–14.60)	11.60 (9.00–14.60)	11.60 (9.20–14.90)	0.003
DBIL, μmol/L	2.00 (1.50–2.90)	2.00 (1.40–2.60)	2.00 (1.40–2.60)	1.90 (1.40–2.60)	1.90 (1.40–2.60)	< 0.001
BUN, mmol/L	4.76 ± 1.63	4.56 ± 1.53	4.53 ± 1.39	4.58 ± 1.25	4.50 ± 1.20	< 0.001
CR, mmol/L	78.00 (68.00–93.00)	77.00 (67.00–92.00)	80.00 (68.00–94.00)	84.00 (72.00–95.00)	87.00 (76.00–96.00)	< 0.001
UA, μmol/L	284.90 ± 94.09	275.54 ± 89.22	284.15 ± 88.02	304.56 ± 86.09	317.18 ± 83.47	< 0.001
FPG, mmol/L	5.12 ± 0.65	5.16 ± 0.74	5.24 ± 0.82	5.27 ± 0.87	5.28 ± 1.03	< 0.001
TC, mmol/L	4.58 ± 0.75	4.57 ± 0.72	4.61 ± 0.74	4.62 ± 0.77	4.66 ± 0.74	< 0.001
TG, mmol/L	0.99 (0.77–1.35)	1.02 (0.79–1.40)	1.11 (0.84–1.54)	1.23 (0.92–1.75)	1.40 (1.02–2.02)	< 0.001
HDL-C, mmol/L	1.53 ± 0.37	1.51 ± 0.35	1.48 ± 0.36	1.40 ± 0.34	1.33 ± 0.34	< 0.001
LDL-C, mmol/L	2.23 (1.89–2.56)	2.24 (1.91–2.57)	2.30 (1.95–2.63)	2.32 (1.96–2.66)	2.40 (2.05–2.71)	< 0.001

Values are n(%) or mean ± SD

*Abbreviations*: *BMI* body mass index, *BUN* blood urea nitrogen, *Cr* creatinine, *UA* uric acid, *FPG* fasting plasma glucose, *TC* total cholesterol, *TG* triglyceride, *HDL-C* high-density lipoprotein cholesterol, *LDL-C* low-density lipoprotein cholesterol, *ALP* Alkaline phosphatase, *GGT* gamma-glutamyl transferase, *ALT* alanine aminotransferase, *AST* aspartate aminotransferase, *TP* Total Protein, *ALB* albumin, *GLB* globulin, *TB* Total bilirubin, *DBIL* Direct bilirubin, *DBP* diastolic blood pressure, *SBP* systolic blood pressure, *FPG* fasting plasma glucose

Table 2 shows the association between ALT/AST ratio and the risk of NAFLD in nonobese Chinese individuals. In the crude model, there was a positive correlation of the ALT/AST ratio with NAFLD risk, and the HRs corresponding to the quintile of the ALT/AST ratio were 1, 2.00 (1.62, 2.47), 3.01 (2.47, 3.67), 4.82 (3.99, 5.81) and 7.81 (6.52, 9.36) (*P* for trend< 0.001) for NAFLD risk. In the model adjusted for sex and age (Model I), the core outcome of the association between the ALT/AST ratio and NAFLD risk did not change significantly (adjusted HRs [aHRs]: 2.61, 95% CI: 2.48, 2.76). In the multifactor model (Model II), age, sex, DBP, ALP, SBP, GGT, BMI, GLB, height, TB, CR, DBIL, BUN, TG, UA, FPG and HDL-C were further adjusted according to STROBE recommendations. It can be observed when the ALT/AST ratio increased by 1, the risk of NAFLD increased by 2.61 times (aHRs: 2.61, 95% CI: 2.48, 2.76), and the increasing trend between the quintile of the ALT/AST ratio and NAFLD risk remained unchanged (*P* for trend< 0.001). Following further adjustments based on the recommended methodology (Model III), the results were similar to model II, with no significant changes in the magnitude or direction of the core results. Finally, after adjusting all non-collinear variables (Model IV), the ALT/AST ratio was still positively correlated with NAFLD risk (aHRs: 2.10, 95% CI: 1.88, 2.36). The aHRs corresponding to the quintiles of the ALT/AST ratio were 1, 1.63 (1.30, 2.04), 2.07 (1.65, 2.60), 2.84 (2.33, 3.48) and 3.49 (2.78, 4.39) (*P* for trend< 0.001).

**Table 2:** Association between ALT/AST ratio and NAFLD in different models

	Crude Model	Model I	Model II	Model III	Model IV
	HRs (95%CI)	aHRs (95%CI)	aHRs (95%CI)	aHRs (95%CI)	aHRs (95%CI)
ALT/AST ratio	2.58 (2.44, 2.72)	2.61 (2.48, 2.76)	2.21 (1.97, 2.48)	2.11 (1.88, 2.36)	2.10 (1.88, 2.36)
ALT/AST ratio (Quintile)					
Quintile 1	Ref	Ref	Ref	Ref	Ref
Quintile 2	2.00 (1.62, 2.47)	2.01 (1.63, 2.48)	1.66 (1.33, 2.08)	1.63 (1.30, 2.04)	1.63 (1.30, 2.04)
Quintile 3	3.01 (2.47, 3.67)	3.04 (2.49, 3.72)	2.24 (1.82, 2.76)	2.06 (1.64, 2.60)	2.07 (1.65, 2.60)
Quintile 4	4.82 (3.99, 5.81)	4.90 (4.07, 5.90)	2.99 (2.46, 3.65)	2.86 (2.33, 3.50)	2.84 (2.33, 3.48)
Quintile 5	7.81 (6.52, 9.36)	7.97 (6.65, 9.55)	3.81 (3.14, 4.62)	3.51 (2.82, 4.37)	3.49 (2.78, 4.39)
*P* for trend	< 0.001	< 0.001	< 0.001	< 0.001	< 0.001

Crude model adjusted for none

Model I adjusted for sex and age

Model II adjusted for sex, age, GGT, DBIL, TG, HDL-C and BMI

Model III adjusted for sex, age, ALP, GGT, GLB, TB, DBIL, BUN, CR, UA, FPG, TG, HDL-C, Height, BMI, SBP and DBP

Model IV adjusted for sex, age, GGT, ALB, GLB, TB, DBIL, SBP, DBP, ALP, BUN, CR, UA, FPG, TG, HDL-C, LDL-C, Height and BMI

*Abbreviations*: *CI* confidence interval, *aHRs* adjusted hazard ratios; other abbreviations as in Table 1

To further investigate the association of the ALT/AST ratio with NAFLD risk, the researchers conducted a stratified analysis and found that the ALT/AST ratio was associated with a reduced risk for NAFLD at age ≥ 70 years, in contrast with other age groups in the assigned subgroup. Additionally, when the ALT/AST ratio increased, men (aHRs: 2.22, 95% CI: 1.81, 2.71) had a higher risk of NAFLD than women (aHRs: 2.01, 95% CI: 1.70, 2.37), and this association also existed in people with high blood pressure, high blood lipids and hyperglycaemia (Table 3). However, in those with high GGT levels, the ALT/AST ratio increased but the NAFLD risk decreased. The detailed information of the above statistical analysis steps using imputation data and original data are summarized in Additional file 1: Supplementary Tables 4, 5 and 6.

**Table 3:** Subgroup analysis by stratified cox regression model

Variables	NAFLD	aHRs (95%CI)
Age (years)		
≥ 70	193 (8.99%)	1.83 (1.11, 3.02)
≥ 60, < 70	161 (7.50%)	2.49 (1.54, 4.03)
≥ 50, < 60	331 (15.42%)	2.48 (1.67, 3.67)
≥ 40, < 50	513 (23.89%)	2.15 (1.63, 2.86)
≥ 30, < 40	600 (27.95%)	1.88 (1.51, 2.33)
< 30	349 (16.26%)	2.45 (1.38, 4.36)
Sex		
Female	937 (43.64%)	2.01 (1.70, 2.37)
Male	1210 (56.36%)	2.22 (1.81, 2.71)
SBP, mmHg		
≥ 140	469 (21.85%)	2.55 (1.94, 3.35)
< 140	1677 (78.15%)	2.03 (1.78, 2.32)
DBP, mmHg		
≥ 90	327 (15.24%)	2.46 (1.76, 3.45)
< 90	1819 (84.76%)	2.03 (1.79, 2.32)
GGT, U/L		
≥ 40	736 (34.28%)	1.64 (1.35, 2.01)
< 40	1411 (65.72%	2.38 (1.84, 3.07)
FPG, mmol/L		
< 3.9	1 (0.05%)	NA
≥ 3.9, < 6.1	1853 (86.31%)	2.08 (1.83, 2.37)
≥ 6.1	293 (13.65%)	2.39 (1.50, 3.82)
TC, mmol/L		
> 5.2	609 (28.37%)	2.41 (1.86, 3.13)
≤ 5.2	1538 (71.63%)	2.04 (1.79, 2.33)
TG, mmol/L		
≥ 1.7	1043 (48.58%)	2.17 (1.72, 2.72)
< 1.7	1104 (51.42%)	2.12 (1.73, 2.60)

*Abbreviations*: *CI* confidence interval, *aHRs* adjusted hazard ratios; other abbreviations as in Table 1. The above model adjusted for sex, age, GGT, ALB, GLB, TB, DBIL, SBP, DBP, ALP, BUN, CR, UA, FPG, TG, HDL-C, LDL-C, Height and BMI. Note: In each case, the model is not adjusted for the stratification variable

**Fig. 1:**
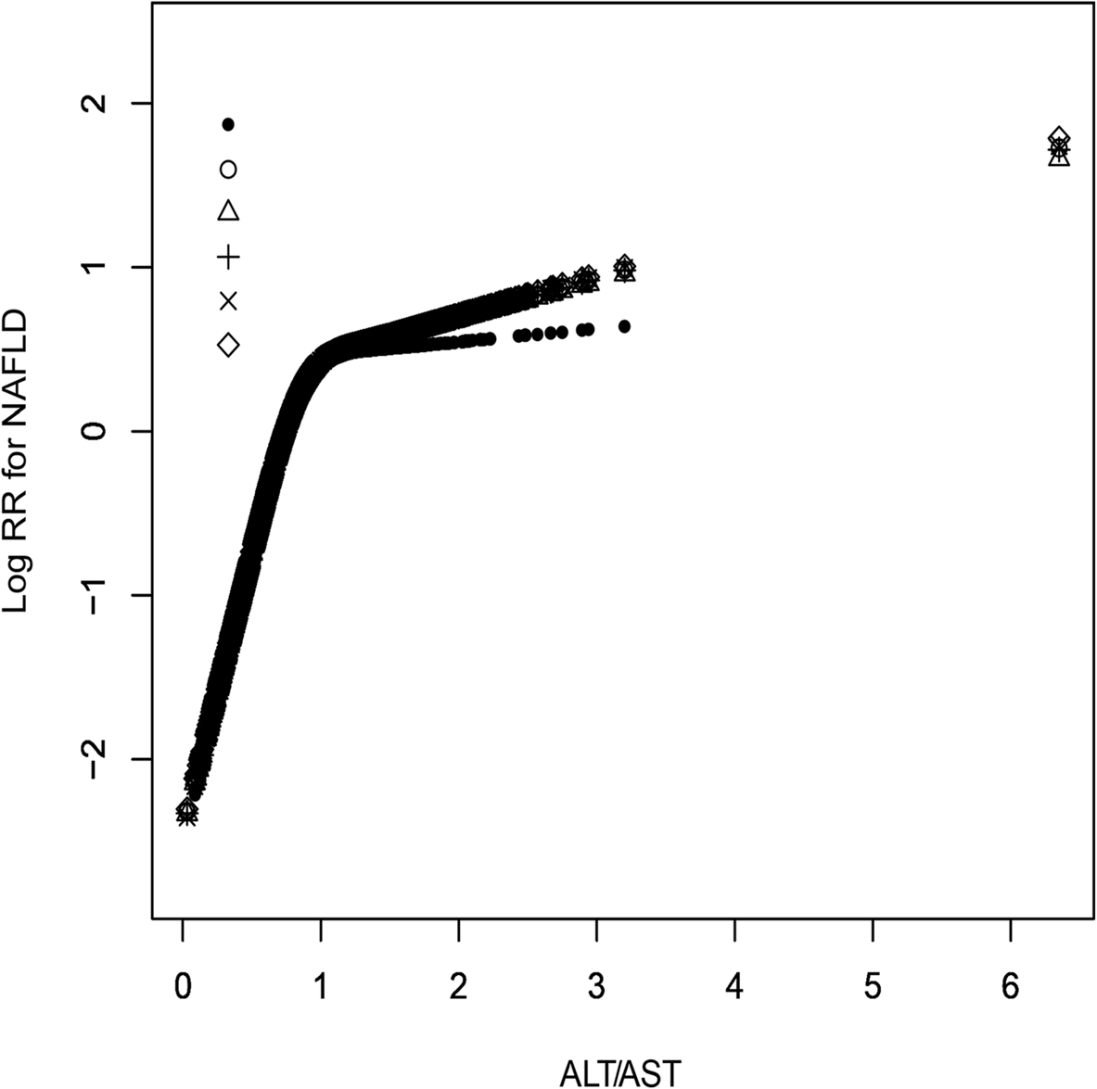
The nonlinear association of ALT/AST ratio with the risk of NAFLD (adjusted for sex, age, GGT, ALB, GLB, TB, DBIL, SBP, DBP, ALP, BUN, CR, UA, FPG, TG, HDL-C, LDL-C, Height and BMI). Different line patterns indicated different data sources (pre- or post imputation). Solid point: original data; Hollow point: pro-imputation 1; Triangle: post-imputation 2; Plus sign: post-imputation 3; Multiplication sign: post-imputation 4; Rhombus: post-imputation 5

**Fig. 2:**
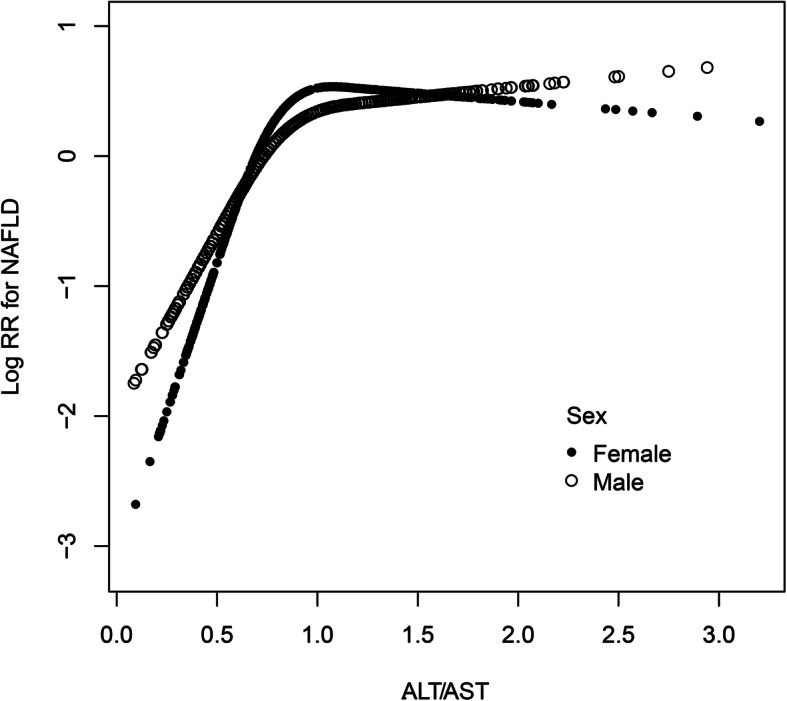
The nonlinear association of ALT/AST ratio with the risk of NAFLD in different gender (adjusted for age, GGT, ALB, GLB, TB, DBIL, SBP, DBP, ALP, BUN, CR, UA, FPG, TG, HDL-C, LDL-C, Height and BMI)

The GAM was used to simulate whether there was a nonlinear correlation of the ALT/AST ratio with the risk of NAFLD. Regression splines suggested that when the ALT/AST ratio was approximately 1 (Fig. 1), there might be a saturation effect of NAFLD risk. Through the recursive algorithm, the inflection point of the ALT/AST ratio was calculated to be 0.93. Table 4 shows the results of the analysis of the original data, the result shows that when the ALT/AST ratio was ≤0.93, the aHRs was 14.41 (8.91, 23.30); however, when the ALT/AST ratio was > 0.93, the aHRs was 1.01 (0.78, 1.30). Additionally, the nonlinear association between ALT/AST ratio and NAFLD risk between different genders was further analyzed. As expected, there was not only a nonlinear association between ALT/AST ratio and NAFLD risk by gender (Fig. 2), but also a saturation effect, in which the saturation effect of ALT/AST ratio on event NAFLD was 1.22 in men and 0.89 in women. The details are summarized in Supplementary Table 7 (Additional file 1).

**Table 4:** The result of the two-piecewise linear regression model

	β	(aHRs,95%CI)	*P*-value
*Total*			
Fitting model by standard linear regression	0.74	2.09 (1.78, 2.46)	< 0.001
Fitting model by two-piecewise linear regression			
The inflection point of ALT/AST ratio		0.93	
≤ 0.93	2.67	14.41 (8.91, 23.30)	< 0.001
> 0.93	0.01	1.01 (0.78, 1.30)	0.939
*Male*			
Fitting model by standard linear regression	0.71	2.02 (1.60, 2.56)	< 0.001
Fitting model by two-piecewise linear regression			
The inflection point of ALT/AST ratio		1.22	
≤ 1.22	1.48	4.39 (2.97, 6.49)	< 0.001
> 1.22	−0.42	0.65 (0.37, 1.15)	0.143
*Female*			
Fitting model by standard linear regression	0.70	2.01 (1.60, 2.54)	< 0.001
Fitting model by two-piecewise linear regression			
The inflection point of ALT/AST ratio		0.89	
≤0.89	3.47	32.01 (14.18, 72.25)	< 0.001
> 0.89	−0.19	0.83 (0.57, 1.21)	0.326

Adjust for: sex, age, GGT, ALB, GLB, TB, DBIL, SBP, DBP, ALP, BUN, CR, UA, FPG, TG, HDL-C, LDL-C, height and BMI

## Discussion

This study shows that the ALT/AST ratio was significantly and independently associated with NAFLD risk in nonobese Chinese individuals (aHRs: 2.10, 95% CI: 1.88, 2.36), and this association was independent of other metabolic changes.

ALT and AST are mainly found in hepatocytes. When hepatocytes are damaged, elevated levels of ALT and AST often indicate liver injury. The ALT/AST ratio, as a composite index, was thought to be associated with hepatic fat infiltration and can be used to identify liver steatosis [20, 21]. In several recent cross-sectional studies, researchers have found that the ALT/AST ratio was independently related to NAFLD. A Korean study that surveyed 10,724 healthy people was the first to find a correlation between the ALT/AST ratio and NAFLD [23]. Later, a study in Taiwan conducted by Lin et al. analyzed the characteristics of 1354 patients infected with the hepatitis C virus and found that the ALT/AST ratio may be the main cause of hepatic steatosis [24]. Similar results have been reported once in China [22]. In a recent study, Lu et al. also found a correlation of the ALT/AST ratio with NAFLD risk in 1222 adolescents and children [25], which was similar to the results of age stratification in this research. Although these studies emphasize the correlation of the ALT/AST ratio with NAFLD risk, they were all cross-sectional in design, and the sample sizes were relatively small (1222, 1354, 6926 and 10,724). The participants were not all nonobese, and a nonlinear correlation of the ALT/AST ratio with the NAFLD risk and differences between different subgroups were not found. Therefore, the association of the ALT/AST ratio with NAFLD risk still needs to be further explored. Based on previous studies, this study adopted a longitudinal cohort design and continued to expand the sample size. After strict statistical screening and adjustment, the obtained evidence was consistent with previous studies. Furthermore, there is a nonlinear correlation between the ALT/AST ratio and NAFLD risk. The inflexion point showing a saturation effect was present on the right side, which means when the ALT/AST ratio was > 0.93, the NAFLD risk hardly increased. The saturation effect between ALT/AST ratio and NAFLD risk may be related to the plateau of skeletal muscle insulin sensitivity [34]. It is well known that skeletal muscle insulin resistance (IR) is the main pathophysiological mechanism of NAFLD [35, 36], and ALT/AST ratio is considered to be the best predictor of IR in Asian population [37, 38]. In a recent study by Bril et al., they found that ALT and AST were positively correlated with the accumulation of intrahepatic triglyceride (IHTG), and when IHTG reached 4.2%, the IR of skeletal muscle would reach a plateau [35], that is, whether IHTG continued to increase or not would not cause any change in the insulin sensitivity of skeletal muscle. This may be the reason why the risk of NAFLD tends to flatten when the ALT/AST ratio is more than 0.93 (1.22 in men and 0.89 in women), and the real pathophysiological mechanism needs to be further studied in the future. Besides, different genders also have the characteristics of nonlinear correlation and saturation effects. When the male ALT/AST ratio > 1.22 and the female ALT/AST ratio > 0.98, the risk of NAFLD hardly increased. More interestingly, this nonlinear trend seems to be U-shaped among women.

The subgroup analysis also found that compared with other age groups, when the ALT/AST ratio increased, the risk of NAFLD decreased in people over 70 years old, and a similar effect was also noted in other studies; this finding may be associated with reduced ALT levels in older age groups [39]. Additionally, when the ALT/AST ratio increased, the NAFLD risk of men was higher than that of women. This association also existed in people with high blood pressure, high blood lipids and hyperglycaemia. However, in people with high levels of GGT, the ratio of ALT/AST increased but the NAFLD risk decreases. Similar views have been expressed by Oh et al., who showed in a recent study that the reference range for GGT level was dose-dependent on the risk of NAFLD [19].

There has long been a consensus among most people regarding the correlation of obesity with NAFLD risk, but data regarding the risk of NAFLD in nonobese people are limited. In previous studies, researchers found that NAFLD in nonobese people was independently correlated with risk factors such as age, height, BMI, TG, TC, LDL-C, UA, GGT, ALT, AST, DBIL, and blood glucose metabolism [26, 40, 41]. In this study, through the follow-up of 12,127 nonobese participants with no NAFLD at baseline, it was proved that the ALT/AST ratio was an independent risk factor for new-onset NAFLD in nonobese people and that the NAFLD risk increased as the ALT/AST ratio gradually increased (*P* for trend< 0.001).

Some possible reasons may explain why a higher ALT/AST ratio could easily lead to new-onset NAFLD. (i) A higher ALT/AST ratio has been associated with hepatic fat infiltration and hepatic steatosis [20, 21]; (ii) in the Asian population, the ALT/AST ratio was considered to be a reliable marker of insulin resistance (IR) [37, 38, 42], while IR was a key pathophysiological factor for NAFLD [43]; and (iii) a higher ALT/AST ratio is closely related to inflammation in the liver [24], and chronic inflammation could further induce steatosis and IR in the liver [1, 24]. In summary, a higher ALT/AST ratio may lead to chronic inflammation of the liver, IR and steatosis of the liver, which further leads to the occurrence of NAFLD.

The ALT/AST ratio is a cheap and easily measured clinical indicator, and the results of this study enrich the latest evidence for new-onset NAFLD in nonobese individuals. Based on the results of the current research, more attention should be paid to the evaluation of the ALT/AST ratio in nonobese individuals, especially in people with abnormal blood pressure, blood glucose or blood lipids, people with normal GGT levels, and men.

### Study strengths and limitations

Several major strengths of this study lie in the inclusion of relatively strict statistical screening and adjustment parameters, the adoption of a longitudinal cohort design, the relatively large sample size, the exploration of the nonlinear association between ALT/AST ratio and NAFLD risk and the performance of a hierarchical analysis.

Several limitations are also noteworthy: (i) the conclusions of this study are only applicable to the Chinese population, and the ALT/AST ratio and NAFLD risk were different by sex and age; (ii) although ultrasound has high accuracy in screening for NAFLD, it is still not comparable to the gold standard liver biopsy, which may mean that the true incidence of NAFLD was be underestimated; and (iii) as with other observational studies, this research could not eliminate confounding factors that actually exist but could not be measured.

## Conclusion

In summary, a higher ALT/AST ratio was independently associated with new-onset NAFLD in this longitudinal cohort study during the 5-year follow-up of Chinese nonobese people, and this independent association was more significant among people with high blood pressure, high blood lipids, hyperglycaemia and in men. The findings in the present work provide a simple and convenient detection index for the early evaluation of the incidence of NAFLD in nonobese Chinese individuals and identification of nonobese high-risk people who are prone to NAFLD, which is conducive to the early initiation of intervention measures in nonobese people.

## Supplementary Information

**Additional file 1:** **Supplement Table 1.** The description of missing data. **Supplementary Table 2.** Sensitivity comparative analysis between pre-imputation and post-imputation. **Supplementary Table 3.** Collinearity diagnostics steps. **Supplementary Table 4.** Results of multivariate linear regression among original data and post-imputation data. **Supplementary Table 5.** Results of multivariate linear regression among original data and post-imputation data in different genders. **Supplementary Table 6.** Subgroup analysis by stratified cox regression model. **Supplementary Table 7.** nonlinearity among original data and post-imputation data.

## Data Availability

The datasets analysed during the current study were acquired from the DRYAD database [https://datadryad.org].

## Abbreviations

STROBE: Strengthening the Reporting of Observational Studies in Epidemiology
IR: Insulin resistance
ALT/AST ratio: Alanine aminotransferase/aspartate aminotransferase ratio
HRs: Hazard ratio
BMI: Body mass index
GLB: Globulin
ALT: Alanine aminotransferase
CI: Confidence interval
FPG: Fasting plasma glucose
DBP: Diastolic blood pressure
NAFLD: Non-alcoholic fatty liver disease
ALP: Alkaline phosphatase
TG: Triglyceride
BUN: Blood urea nitrogen
TP: Total protein
GAM: Generalized additive model
Cr: Creatinine
GGT: Gamma-glutamyl transferase
LDL-C: Low-density lipoprotein cholesterol
UA: Uric acid
TC: Total cholesterol
TB: Total bilirubin
HDL-C: High-density lipoprotein cholesterol
SBP: Systolic blood pressure
DBIL: Direct bilirubin
AST: Aspartate aminotransferase
