# Supplementary material for: Association between the alanine aminotransferase/aspartate aminotransferase ratio and new-onset non-alcoholic fatty liver disease in a nonobese Chinese population: a population-based longitudinal study
Source: Lipids Health Dis. 2020 Nov 25;19:245. doi: 10.1186/s12944-020-01419-z (PMC7690093; doi:10.1186/s12944-020-01419-z)
Supplement: Supplementary file 1 — Additional file 1: Supplement Table 1. The description of missing data. Supplementary Table 2. Sensitivity comparative analysis between pre-imputation and post-imputation. Supplementary Table 3. Collinearity diagnostics steps. Supplementary Table 4. Results of multivariate linear regression among original data and post-imputation data. Supplementary Table 5. Results of multivariate linear regression among original data and post-imputation data in different genders. Supplementary Table 6. Subgroup analysis by stratified cox regression model. Supplementary Table 7. nonlinearity among original data and post-imputation data. [file 12944_2020_1419_MOESM1_ESM.docx]

Supplement Table 1: The description of missing data.

| Variables | Non-missing | Missing |
| --- | --- | --- |
| Sex | 12127 | 0 |
| Age | 12127 | 0 |
| Weight | 12127 | 0 |
| Height | 12127 | 0 |
| BMI | 12127 | 0 |
| SBP | 12111 | 16 |
| DBP | 12111 | 16 |
| ALP | 12127 | 0 |
| GGT | 12125 | 2 |
| ALT | 12127 | 0 |
| AST | 12127 | 0 |
| ALT/AST | 12127 | 0 |
| TP | 10748 | 1379 |
| ALB | 10748 | 1379 |
| GLB | 10748 | 1379 |
| TB | 8957 | 3170 |
| DBIL | 7546 | 4581 |
| BUN | 12127 | 0 |
| CR | 12127 | 0 |
| UA | 12127 | 0 |
| FPG | 12127 | 0 |
| TC | 12127 | 0 |
| TG | 12127 | 0 |
| HDL-C | 12127 | 0 |
| LDL-C | 12127 | 0 |

Supplementary Table 2: Sensitivity comparative analysis between pre-imputation and post-imputation.

|  | original data | imputation 1 | imputation 2 | imputation 3 | imputation 4 | imputation 5 | *P*-value |
| --- | --- | --- | --- | --- | --- | --- | --- |
| N | 12127 | 12127 | 12127 | 12127 | 12127 | 12127 |  |
| Sex |  |  |  |  |  |  | 1.000 |
| Female | 5481 (45.20%) | 5481 (45.20%) | 5481 (45.20%) | 5481 (45.20%) | 5481 (45.20%) | 5481 (45.20%) |  |
| Male | 6646 (54.80%) | 6646 (54.80%) | 6646 (54.80%) | 6646 (54.80%) | 6646 (54.80%) | 6646 (54.80%) |  |
| Age | 43.29±14.95 | 43.29±14.95 | 43.29±14.95 | 43.29±14.95 | 43.29±14.95 | 43.29±14.95 | 1.000 |
| Weight | 59.91±8.54 | 59.91±8.54 | 59.91±8.54 | 59.91±8.54 | 59.91±8.54 | 59.91±8.54 | 1.000 |
| Height | 1.66±0.08 | 1.66±0.08 | 1.66±0.08 | 1.66±0.08 | 1.66±0.08 | 1.66±0.08 | 1.000 |
| BMI | 21.61±2.04 | 21.61±2.04 | 21.61±2.04 | 21.61±2.04 | 21.61±2.04 | 21.61±2.04 | 1.000 |
| SBP | 122.41±16.94 | 122.40±16.93 | 122.41±16.94 | 122.41±16.94 | 122.41±16.93 | 122.43±16.95 | 1.000 |
| DBP | 73.81±10.39 | 73.80±10.39 | 73.80±10.39 | 73.81±10.39 | 73.81 ±10.39 | 73.82±10.40 | 1.000 |
| GGT | 22 (16-31) | 22 (16-31) | 22 (16-31) | 22 (16-31) | 22 (16-31) | 22 (16-31) | 1.000 |
| TP | 73.85±4.26 | 73.86±4.22 | 73.86±4.22 | 73.86±4.22 | 73.86±4.22 | 73.86±4.22 | 1.000 |
| ALB | 44.54±2.77 | 44.55±2.99 | 44.55±2.99 | 44.55±2.99 | 44.55±2.99 | 44.55±2.99 | 1.000 |
| GLB | 29.32±3.98) | 29.31±3.99 | 29.31±3.99 | 29.31±3.99 | 29.31±3.99 | 29.31 (3.99) | 1.000 |
| TB | 12.23±5.06 | 12.32±5.06 | 12.34±5.07 | 12.31±5.05 | 12.37±5.03 | 12.39±5.02 | 0.274 |
| DBIL | 2.19±1.20 | 2.23±1.20 | 2.23±1.20 | 2.25±1.20 | 2.23±1.19 | 2.23±1.19 | 0.071 |
| ALP | 72.35±23.22 | 72.35±23.22 | 72.35±23.22 | 72.35±23.22 | 72.35±23.22 | 72.35±23.22 | 1.000 |
| AST | 23.04±9.53 | 23.04±9.53 | 23.04±9.53 | 23.04±9.53 | 23.04±9.53 | 23.04±9.53 | 1.000 |
| ALT | 16 (12-23) | 16 (12-23) | 16 (12-23) | 16 (12-23) | 16 (12-23) | 16 (12-23) | 1.000 |
| BUN | 4.58±1.41 | 4.58±1.41 | 4.58±1.41 | 4.58±1.41 | 4.58±1.41 | 4.58±1.41 | 1.000 |
| ALT/AST | 0.84±0.31 | 0.84±0.31 | 0.84±0.31 | 0.84±0.31 | 0.84±0.31 | 0.84±0.31 | 1.000 |
| UA | 293.41±89.52 | 293.41±89.52 | 293.41±89.52 | 293.41±89.52 | 293.41±89.52 | 293.41±89.52 | 1.000 |
| CR | 83.55±26.40 | 83.55±26.40 | 83.55±26.40 | 83.55±26.40 | 83.55±26.40 | 83.55±26.40 | 1.000 |
| FPG | 5.21±0.83 | 5.21±0.83 | 5.21±0.83 | 5.21±0.83 | 5.21±0.83 | 5.21±0.83 | 1.000 |
| TC | 4.61±0.74 | 4.61±0.74 | 4.61±0.74 | 4.61±0.74 | 4.61±0.74 | 4.61±0.74 | 1.000 |
| TG | 1.14 (0.85-1.60) | 1.14 (0.85-1.60) | 1.14 (0.85-1.60) | 1.14 (0.85-1.60) | 1.14 (0.85-1.60) | 1.14 (0.85-1.60) | 1.000 |
| HDL-C | 1.45±0.36 | 1.45±0.36 | 1.45±0.36 | 1.45±0.36 | 1.45±0.36 | 1.45±0.36 | 1.000 |
| LDL-C | 2.27±0.47 | 2.27±0.47 | 2.27±0.47 | 2.27±0.47 | 2.27±0.47 | 2.27±0.47 | 1.000 |

Values are n(%) or mean ± SD; Abbreviations: BMI: body mass index; BUN: blood urea nitrogen; Cr: creatinine; UA: uric acid; FPG: fasting plasma glucose; TC: total cholesterol; TG: triglyceride; HDL-C: high-density lipoprotein cholesterol; LDL-C: low-density lipoprotein cholesterol; ALP: Alkaline phosphatase; GGT: gamma-glutamyl transferase; ALT: alanine aminotransferase; AST: aspartate aminotransferase; TP: Total Protein; ALB: albumin; GLB: globulin; TB: Total bilirubin; DBIL: Direct bilirubin; DBP: diastolic blood pressure; SBP: systolic blood pressure; FPG: fasting plasma glucose.

Supplementary Table 3: Collinearity diagnostics steps.

|  | Step 1 | Step 2 | Step 3 | Step 4 |
| --- | --- | --- | --- | --- |
| GGT | 1.3 | 1.3 | 1.3 | 1.3 |
| TP | 18109377.5 | NA | NA | NA |
| ALB | 8886690.8 | 1.2 | 1.2 | 1.2 |
| GLB | 16224400 | 1.1 | 1.1 | 1.1 |
| TB | 2 | 2 | 2 | 2 |
| DBIL | 2 | 2 | 2 | 2 |
| SBP | 2.5 | 2.5 | 2.5 | 2.5 |
| DBP | 2.2 | 2.2 | 2.2 | 2.2 |
| Sex | 1 | 1 | 1 | 1 |
| Age | 1.1 | 1.1 | 1.1 | 1.1 |
| ALP | 1.2 | 1.2 | 1.2 | 1.2 |
| ALT/AST | 1.3 | 1.3 | 1.3 | 1.3 |
| BUN | 1.5 | 1.5 | 1.5 | 1.4 |
| CR | 1.5 | 1.5 | 1.5 | 1.5 |
| UA | 1.7 | 1.7 | 1.7 | 1.7 |
| FPG | 2.1 | 2.1 | 2.1 | 2.1 |
| TC | 6.4 | 6.4 | 6.4 | NA |
| TG | 2.3 | 2.3 | 2.3 | 1.4 |
| HDL-C | 2.5 | 2.5 | 2.5 | 1.3 |
| LDL-C | 4.7 | 4.7 | 4.7 | 1.1 |
| Weight | 242.8 | 242.8 | NA | NA |
| Height | 104.2 | 104.2 | 1.4 | 1.4 |
| BMI | 107.4 | 107.4 | 1.3 | 1.3 |

VIF = 1/(1-R^2^).

Supplementary Table 4: Results of multivariate linear regression among original data and post-imputation data.

|  | Original data | | Post-imputation 1 | | Post-imputation 2 | | Post-imputation 3 | | Post-imputation 4 | | Post-imputation 5 | |
| --- | --- | --- | --- | --- | --- | --- | --- | --- | --- | --- | --- | --- |
|  | β | aHRs (95%CI) | β | aHRs (95%CI) | β | aHRs (95%CI) | β | aHRs (95%CI) | β | aHRs (95%CI) | β | aHRs (95%CI) |
| Crude Model | | | | | | | | | | | | |
| ALT/AST ratio | 0.95 | 2.58 (2.44, 2.72)* | 0.95 | 2.58 (2.44, 2.72)* | 0.95 | 2.58 (2.44, 2.72)* | 0.95 | 2.58 (2.44, 2.72)* | 0.95 | 2.58 (2.44, 2.72)* | 0.95 | 2.58 (2.44, 2.72)* |
| ALT/AST ratio (Quintile) | | | | | | | | | | | | |
| Q1 | Ref | Ref | Ref | Ref | Ref | Ref | Ref | Ref | Ref | Ref | Ref | Ref |
| Q2 | 0.69 | 2.00 (1.62, 2.47)* | 0.69 | 2.00 (1.62, 2.47)* | 0.69 | 2.00 (1.62, 2.47)* | 0.69 | 2.00 (1.62, 2.47)* | 0.69 | 2.00 (1.62, 2.47)* | 0.69 | 2.00 (1.62, 2.47)* |
| Q3 | 1.10 | 3.01 (2.47, 3.68)* | 1.10 | 3.01 (2.47, 3.68)* | 1.10 | 3.01 (2.47, 3.68)* | 1.10 | 3.01 (2.47, 3.68)* | 1.10 | 3.01 (2.47, 3.68)* | 1.10 | 3.01 (2.47, 3.68)* |
| Q4 | 1.57 | 4.82 (4.00, 5.81)* | 1.57 | 4.82 (4.00, 5.81)* | 1.57 | 4.82 (4.00, 5.81)* | 1.57 | 4.82 (4.00, 5.81)* | 1.57 | 4.82 (4.00, 5.81)* | 1.57 | 4.82 (4.00, 5.81)* |
| Q5 | 2.06 | 7.81 (6.52, 9.36)* | 2.06 | 7.81 (6.52, 9.36)* | 2.06 | 7.81 (6.52, 9.36)* | 2.06 | 7.81 (6.52, 9.36)* | 2.06 | 7.81 (6.52, 9.36)* | 2.06 | 7.81 (6.52, 9.36)* |
| *P* for trend |  | <0.001 |  | <0.001 |  | <0.001 |  | <0.001 |  | <0.001 |  | <0.001 |
| Model I | | | | | | | | | | | | |
| ALT/AST ratio | 0.96 | 2.61 (2.48, 2.76)* | 0.96 | 2.61 (2.48, 2.76)* | 0.96 | 2.61 (2.48, 2.76)* | 0.96 | 2.61 (2.48, 2.76)* | 0.96 | 2.61 (2.48, 2.76)* | 0.96 | 2.61 (2.48, 2.76)* |
| ALT/AST ratio (Quintile) | | | | | | | | | | | | |
| Q1 | Ref | Ref | Ref | Ref | Ref | Ref | Ref | Ref | Ref | Ref | Ref | Ref |
| Q2 | 0.70 | 2.01 (1.63, 2.49)* | 0.70 | 2.01 (1.63, 2.49)* | 0.70 | 2.01 (1.63, 2.49)* | 0.70 | 2.01 (1.63, 2.49)* | 0.70 | 2.01 (1.63, 2.49)* | 0.70 | 2.01 (1.63, 2.49)* |
| Q3 | 1.11 | 3.04 (2.49, 3.72)* | 1.11 | 3.04 (2.49, 3.72)* | 1.11 | 3.04 (2.49, 3.72)* | 1.11 | 3.04 (2.49, 3.72)* | 1.11 | 3.04 (2.49, 3.72)* | 1.11 | 3.04 (2.49, 3.72)* |
| Q4 | 1.59 | 4.90 (4.07, 5.91)* | 1.59 | 4.90 (4.07, 5.91)* | 1.59 | 4.90 (4.07, 5.91)* | 1.59 | 4.90 (4.07, 5.91)* | 1.59 | 4.90 (4.07, 5.91)* | 1.59 | 4.90 (4.07, 5.91)* |
| Q5 | 2.08 | 7.97 (6.65, 9.55)* | 2.08 | 7.97 (6.65, 9.55)* | 2.08 | 7.97 (6.65, 9.55)* | 2.08 | 7.97 (6.65, 9.55)* | 2.08 | 7.97 (6.65, 9.55)* | 2.08 | 7.97 (6.65, 9.55)* |
| *P* for trend |  | <0.001 |  | <0.001 |  | <0.001 |  | <0.001 |  | <0.001 |  | <0.001 |
| Model II | | | | | | | | | | | | |
| ALT/AST ratio | 0.85 | 2.33 (2.04, 2.67)* | 0.75 | 2.17 (1.98, 2.38)* | 0.80 | 2.22 (2.02, 2.44)* | 0.77 | 2.16 (1.98, 2.36)* | 0.78 | 2.19 (2.00, 2.40)* | 0.79 | 2.19 (2.00, 2.41)* |
| ALT/AST ratio (Quintile) | | | | | | | | | | | | |
| Q1 | Ref | Ref | Ref | Ref | Ref | Ref | Ref | Ref | Ref | Ref | Ref | Ref |
| Q2 | 0.47 | 1.61 (1.25, 2.06)* | 0.51 | 1.66 (1.34, 2.05)* | 0.52 | 1.68 (1.36, 2.08)* | 0.52 | 1.68 (1.36, 2.08)* | 0.52 | 1.68 (1.36, 2.07)* | 0.51 | 1.67 (1.35, 2.07)* |
| Q3 | 0.80 | 2.22 (1.75, 2.82)* | 0.81 | 2.24 (1.83, 2.74)* | 0.82 | 2.26 (1.85, 2.76)* | 0.83 | 2.28 (1.87, 2.79)* | 0.80 | 2.22 (1.82, 2.72)* | 0.80 | 2.22 (1.82, 2.71)* |
| Q4 | 1.11 | 3.04 (2.42, 3.80)* | 1.10 | 3.00 (2.48, 3.62)* | 1.09 | 2.98 (2.46, 3.59)* | 1.10 | 3.00 (2.49, 3.63)* | 1.10 | 3.01 (2.49, 3.64)* | 1.08 | 2.94 (2.43, 3.55)* |
| Q5 | 1.35 | 3.85 (3.08, 4.82)* | 1.33 | 3.77 (3.13, 4.54)* | 1.34 | 3.83 (3.18, 4.61)* | 1.35 | 3.86 (3.21, 4.65)* | 1.33 | 3.79 (3.15, 4.57)* | 1.33 | 3.76 (3.13, 4.53)* |
| *P* for trend |  | <0.001 |  | <0.001 |  | <0.001 |  | <0.001 |  | <0.001 |  | <0.001 |
| Model III | | | | | | | | | | | | |
| ALT/AST ratio | 0.76 | 2.13 (1.82, 2.50)* | 0.74 | 2.09 (1.91, 2.30)* | 0.76 | 2.14 (1.94, 2.36)* | 0.72 | 2.06 (1.89, 2.26)* | 0.73 | 2.07 (1.89, 2.26)* | 0.77 | 2.15 (1.95, 2.37)* |
| ALT/AST ratio (Quintile) | | | | | | | | | | | | |
| Q1 | Ref | Ref | Ref | Ref | Ref | Ref | Ref | Ref | Ref | Ref | Ref | Ref |
| Q2 | 0.45 | 1.57 (1.21, 2.03)* | 0.49 | 1.63 (1.31, 2.01)* | 0.51 | 1.66 (1.34, 2.05)* | 0.50 | 1.66 (1.34, 2.05)* | 0.50 | 1.65 (1.33, 2.04)* | 0.48 | 1.62 (1.31, 2.00)* |
| Q3 | 0.63 | 1.88 (1.46, 2.42)* | 0.73 | 2.08 (1.70, 2.55)* | 0.76 | 2.14 (1.75, 2.62)* | 0.75 | 2.12 (1.73, 2.60)* | 0.75 | 2.11 (1.72, 2.58)* | 0.72 | 2.07 (1.69, 2.54)* |
| Q4 | 1.03 | 2.81 (2.21, 3.56)* | 1.05 | 2.88 (2.38, 3.48)* | 1.05 | 2.87 (2.37, 3.47)* | 1.06 | 2.87 (2.37, 3.48)* | 1.07 | 2.92 (2.41, 3.53)* | 1.02 | 2.79 (2.31, 3.38)* |
| Q5 | 1.15 | 3.19 (2.51, 4.05)* | 1.26 | 3.53 (2.92, 4.27)* | 1.28 | 3.61 (2.99, 4.36)* | 1.29 | 3.62 (3.00, 4.37)* | 1.28 | 3.59 (2.97, 4.34)* | 1.27 | 3.55 (2.94, 4.29)* |
| *P* for trend |  | <0.001 |  | <0.001 |  | <0.001 |  | <0.001 |  | <0.001 |  | <0.001 |
| Model IV | | | | | | | | | | | | |
| ALT/AST ratio | 0.74 | 2.09 (1.78, 2.46)* | 0.74 | 2.10 (1.91, 2.30)* | 0.76 | 2.15 (1.95, 2.37)* | 0.72 | 2.06 (1.88, 2.25)* | 0.73 | 2.07 (1.89, 2.26)* | 0.77 | 2.16 (1.95, 2.38)* |
| ALT.AST ratio (Quintile) | | | | | | | | | | | | |
| Q1 | Ref | Ref | Ref | Ref | Ref | Ref | Ref | Ref | Ref | Ref | Ref | Ref |
| Q2 | 0.45 | 1.57 (1.21, 2.04)* | 0.49 | 1.63 (1.32, 2.01)* | 0.51 | 1.66 (1.34, 2.05)* | 0.50 | 1.65 (1.34, 2.05)* | 0.50 | 1.65 (1.33, 2.04)* | 0.48 | 1.62 (1.31, 2.00)* |
| Q3 | 0.64 | 1.90 (1.47, 2.45)* | 0.73 | 2.08 (1.70, 2.55)* | 0.76 | 2.14 (1.75, 2.62)* | 0.75 | 2.11 (1.73, 2.58)* | 0.75 | 2.11 (1.73, 2.59)* | 0.73 | 2.08 (1.70, 2.54)* |
| Q4 | 1.03 | 2.81 (2.21, 3.56)* | 1.05 | 2.87 (2.37, 3.47)* | 1.05 | 2.86 (2.36, 3.46)* | 1.05 | 2.85 (2.35, 3.44)* | 1.07 | 2.91 (2.40, 3.52)* | 1.02 | 2.79 (2.31, 3.38)* |
| Q5 | 1.14 | 3.12 (2.45, 3.97)* | 1.26 | 3.53 (2.92, 4.26)* | 1.28 | 3.61 (2.98, 4.36)* | 1.28 | 3.59 (2.97, 4.33)* | 1.28 | 3.59 (2.97, 4.34)* | 1.27 | 3.55 (2.94, 4.29)* |
| *P* for trend |  | <0.001 |  | <0.001 |  | <0.001 |  | <0.001 |  | <0.001 |  | <0.001 |

**P*<0.05; Abbreviations: CI: confidence interval, aHRs: adjusted hazard ratios; other abbreviations as in Table ​2; Crude model adjusted for none; Model I adjusted for sex and age; Model II adjusted for sex, age, GGT, DBIL, TG, HDL-C and BMI; Model III adjusted for sex, age, ALP, GGT, GLB, TB, DBIL, BUN, CR, UA, FPG, TG, HDL-C, Height, BMI, SBP and DBP; Model IV adjusted for sex, age, GGT, ALB, GLB, TB, DBIL, SBP, DBP, ALP, BUN, CR, UA, FPG, TG, HDL-C, LDL-C, Height and BMI.

Supplementary Table 5: Results of multivariate linear regression among original data and post-imputation data in different genders.

|  | Original data | | Post-imputation 1 | | Post-imputation 2 | | Post-imputation 3 | | Post-imputation 4 | | Post-imputation 5 | |
| --- | --- | --- | --- | --- | --- | --- | --- | --- | --- | --- | --- | --- |
|  | β | aHRs (95%CI) | β | aHRs (95%CI) | β | aHRs (95%CI) | β | aHRs (95%CI) | β | aHRs (95%CI) | β | aHRs (95%CI) |
| *Female* |  |  |  |  |  |  |  |  |  |  |  |  |
| Crude Model |  |  |  |  |  |  |  |  |  |  |  |  |
| ALT/AST ratio | 0.84 | 2.31 (2.14, 2.49)* | 0.84 | 2.31 (2.14, 2.49)* | 0.84 | 2.31 (2.14, 2.49)* | 0.84 | 2.31 (2.14, 2.49)* | 0.84 | 2.31 (2.14, 2.49)* | 0.84 | 2.31 (2.14, 2.49)* |
| ALT/AST ratio (Quintile) | | | | | | | | | | | | |
| Q1 | Ref | Ref | Ref | Ref | Ref | Ref | Ref | Ref | Ref | Ref | Ref | Ref |
| Q2 | 0.77 | 2.15 (1.57, 2.96)* | 0.77 | 2.15 (1.57, 2.96)* | 0.77 | 2.15 (1.57, 2.96)* | 0.77 | 2.15 (1.57, 2.96)* | 0.77 | 2.15 (1.57, 2.96)* | 0.77 | 2.15 (1.57, 2.96)* |
| Q3 | 1.12 | 3.05 (2.25, 4.14)* | 1.12 | 3.05 (2.25, 4.14)* | 1.12 | 3.05 (2.25, 4.14)* | 1.12 | 3.05 (2.25, 4.14)* | 1.12 | 3.05 (2.25, 4.14)* | 1.12 | 3.05 (2.25, 4.14)* |
| Q4 | 1.64 | 5.16 (3.88, 6.87)* | 1.64 | 5.16 (3.88, 6.87)* | 1.64 | 5.16 (3.88, 6.87)* | 1.64 | 5.16 (3.88, 6.87)* | 1.64 | 5.16 (3.88, 6.87)* | 1.64 | 5.16 (3.88, 6.87)* |
| Q5 | 2.12 | 8.35(6.33,11.01)* | 2.12 | 8.35(6.33,11.01)* | 2.12 | 8.35(6.33,11.01)* | 2.12 | 8.35(6.33,11.01)* | 2.12 | 8.35(6.33,11.01)* | 2.12 | 8.35 (6.33, 11.01)* |
| *P* for trend |  | <0.001 |  | <0.001 |  | <0.001 |  | <0.001 |  | <0.001 |  | <0.001 |
| Model I |  |  |  |  |  |  |  |  |  |  |  |  |
| ALT/AST ratio | 0.84 | 2.31 (2.15, 2.49)* | 0.84 | 2.31 (2.15, 2.49)* | 0.84 | 2.31 (2.15, 2.49)* | 0.84 | 2.31 (2.15, 2.49)* | 0.84 | 2.31 (2.15, 2.49)* | 0.84 | 2.31 (2.15, 2.49)* |
| ALT/AST ratio (Quintile) | | | | | | | | | | | | |
| Q1 | Ref | Ref | Ref | Ref | Ref | Ref | Ref | Ref | Ref | Ref | Ref | Ref |
| Q2 | 0.77 | 2.16 (1.57, 2.97)* | 0.77 | 2.16 (1.57, 2.97)* | 0.77 | 2.16 (1.57, 2.97)* | 0.77 | 2.16 (1.57, 2.97)* | 0.77 | 2.16 (1.57, 2.97)* | 0.77 | 2.16 (1.57, 2.97)* |
| Q3 | 1.12 | 3.06 (2.26, 4.15)* | 1.12 | 3.06 (2.26, 4.15)* | 1.12 | 3.06 (2.26, 4.15)* | 1.12 | 3.06 (2.26, 4.15)* | 1.12 | 3.06 (2.26, 4.15)* | 1.12 | 3.06 (2.26, 4.15)* |
| Q4 | 1.65 | 5.19 (3.90, 6.91)* | 1.65 | 5.19 (3.90, 6.91)* | 1.65 | 5.19 (3.90, 6.91)* | 1.65 | 5.19 (3.90, 6.91)* | 1.65 | 5.19 (3.90, 6.91)* | 1.65 | 5.19 (3.90, 6.91)* |
| Q5 | 2.13 | 8.40(6.37,11.08)* | 2.13 | 8.40(6.37,11.08)* | 2.13 | 8.40(6.37,11.08)* | 2.13 | 8.40 (6.37, 11.08)* | 2.13 | 8.40 (6.37, 11.08)* | 2.13 | 8.40 (6.37, 11.08)* |
| *P* for trend |  | <0.001 |  | <0.001 |  | <0.001 |  | <0.001 |  | <0.001 |  | <0.001 |
| Model II |  |  |  |  |  |  |  |  |  |  |  |  |
| ALT/AST ratio | 0.93 | 2.55 (2.10, 3.08)* | 0.74 | 2.09 (1.85, 2.36)* | 0.79 | 2.20 (1.94, 2.50)* | 0.72 | 2.05 (1.83, 2.30)* | 0.75 | 2.11 (1.87, 2.38)* | 0.77 | 2.15 (1.90, 2.44)* |
| ALT/AST ratio (Quintile) | | | | | | | | | | | | |
| Q1 | Ref | Ref | Ref | Ref | Ref | Ref | Ref | Ref | Ref | Ref | Ref | Ref |
| Q2 | 0.67 | 1.96 (1.34, 2.88)* | 0.58 | 1.78 (1.29, 2.45)* | 0.58 | 1.79 (1.30, 2.47)* | 0.60 | 1.83 (1.33, 2.52)* | 0.63 | 1.87 (1.36, 2.58)* | 0.61 | 1.83 (1.33, 2.52)* |
| Q3 | 0.95 | 2.59 (1.79, 3.75)* | 0.76 | 2.13 (1.57, 2.90)* | 0.77 | 2.16 (1.59, 2.94)* | 0.78 | 2.17 (1.60, 2.95)* | 0.78 | 2.18 (1.60, 2.96)* | 0.77 | 2.16 (1.59, 2.93)* |
| Q4 | 1.35 | 3.85 (2.70, 5.48)* | 1.16 | 3.19 (2.39, 4.26)* | 1.15 | 3.17 (2.37, 4.23)* | 1.17 | 3.24 (2.42, 4.32)* | 1.21 | 3.36 (2.51, 4.48)* | 1.16 | 3.19 (2.38, 4.25)* |
| Q5 | 1.61 | 5.01 (3.53, 7.09)* | 1.37 | 3.93 (2.95, 5.22)* | 1.41 | 4.09 (3.08, 5.44)* | 1.39 | 4.03 (3.03, 5.36)* | 1.40 | 4.07 (3.06, 5.41)* | 1.39 | 4.01 (3.02, 5.34)* |
| *P* for trend |  | <0.001 |  | <0.001 |  | <0.001 |  | <0.001 |  | <0.001 |  | <0.001 |
| Model III |  |  |  |  |  |  |  |  |  |  |  |  |
| ALT/AST ratio | 0.73 | 2.08 (1.66, 2.62)* | 0.67 | 1.95 (1.72, 2.22)* | 0.74 | 2.10 (1.84, 2.39)* | 0.65 | 1.92 (1.70, 2.17)* | 0.66 | 1.94 (1.72, 2.19)* | 0.74 | 2.09 (1.84, 2.38)* |
| ALT/AST ratio (Quintile) | | | | | | | | | | | | |
| Q1 | Ref | Ref | Ref | Ref | Ref | Ref | Ref | Ref | Ref | Ref | Ref | Ref |
| Q2 | 0.67 | 1.96 (1.32, 2.92)* | 0.53 | 1.71 (1.24, 2.35)* | 0.55 | 1.73 (1.25, 2.38)* | 0.55 | 1.74 (1.26, 2.40)* | 0.58 | 1.79 (1.30, 2.47)* | 0.56 | 1.75 (1.27, 2.41)* |
| Q3 | 0.85 | 2.35 (1.59, 3.48)* | 0.69 | 2.00 (1.47, 2.72)* | 0.72 | 2.06 (1.52, 2.81)* | 0.71 | 2.04 (1.50, 2.78)* | 0.72 | 2.06 (1.51, 2.81)* | 0.72 | 2.05 (1.50, 2.79)* |
| Q4 | 1.32 | 3.74 (2.58, 5.43)* | 1.10 | 3.01 (2.25, 4.03)* | 1.08 | 2.95 (2.20, 3.95)* | 1.09 | 2.97 (2.22, 3.98)* | 1.15 | 3.16 (2.36, 4.23)* | 1.09 | 2.97 (2.21, 3.98)* |
| Q5 | 1.32 | 3.76 (2.59, 5.46)* | 1.29 | 3.62 (2.71, 4.84)* | 1.33 | 3.79 (2.83, 5.06)* | 1.29 | 3.65 (2.73, 4.87)* | 1.34 | 3.80 (2.84, 5.08)* | 1.33 | 3.79 (2.84, 5.06)* |
| *P* for trend |  | <0.001 |  | <0.001 |  | <0.001 |  | <0.001 |  | <0.001 |  | <0.001 |
| Model IV |  |  |  |  |  |  |  |  |  |  |  |  |
| ALT/AST ratio | 0.70 | 2.01 (1.60, 2.54)* | 0.67 | 1.96 (1.72, 2.23)* | 0.74 | 2.10 (1.84, 2.40)* | 0.66 | 1.93 (1.71, 2.18)* | 0.66 | 1.94 (1.72, 2.19)* | 0.74 | 2.10 (1.84, 2.39)* |
| ALT/AST ratio (Quintile) | | | | | | | | | | | | |
| Q1 | Ref | Ref | Ref | Ref | Ref | Ref | Ref | Ref | Ref | Ref | Ref | Ref |
| Q2 | 0.65 | 1.92 (1.29, 2.86)* | 0.53 | 1.71 (1.24, 2.35)* | 0.55 | 1.73 (1.26, 2.39)* | 0.56 | 1.75 (1.27, 2.42)* | 0.58 | 1.79 (1.30, 2.46)* | 0.56 | 1.75 (1.27, 2.41)* |
| Q3 | 0.85 | 2.35 (1.59, 3.48)* | 0.70 | 2.02 (1.48, 2.75)* | 1.74 | 2.09 (1.53, 2.84)* | 0.73 | 2.07 (1.52, 2.82)* | 0.74 | 2.09 (1.54, 2.85)* | 0.73 | 2.08 (1.52, 2.82)* |
| Q4 | 1.32 | 3.73 (2.57, 5.41)* | 1.10 | 2.99 (2.23, 4.01)* | 1.08 | 2.94 (2.20, 3.95)* | 1.08 | 2.96 (2.21, 3.97)* | 1.15 | 3.15 (2.35, 4.23)* | 1.09 | 2.97 (2.21, 3.98)* |
| Q5 | 1.30 | 3.66 (2.51, 5.32)* | 1.28 | 3.61 (2.70, 4.84)* | 0.33 | 3.78 (2.83, 5.06)* | 1.30 | 3.66 (2.74, 4.89)* | 1.33 | 3.79 (2.84, 5.07)* | 1.33 | 3.80 (2.84, 5.08)* |
| ALT.AST.Q5 |  | <0.001 |  | <0.001 |  | <0.001 |  | <0.001 |  | <0.001 |  | <0.001 |
| *Male* | | | | | | | | | | | | |
| Crude Model |  |  |  |  |  |  |  |  |  |  |  |  |
| ALT/AST ratio | 1.49 | 4.46 (3.91, 5.09)* | 1.49 | 4.46 (3.91, 5.09)* | 1.49 | 4.46 (3.91, 5.09)* | 1.49 | 4.46 (3.91, 5.09)* | 1.49 | 4.46 (3.91, 5.09)* | 1.49 | 4.46 (3.91, 5.09)* |
| ALT/AST ratio (Quintile) | | | | | | | | | | | | |
| Q1 | Ref | Ref | Ref | Ref | Ref | Ref | Ref | Ref | Ref | Ref | Ref | Ref |
| Q2 | 0.64 | 1.89 (1.42, 2.51)* | 0.64 | 1.89 (1.42, 2.51)* | 0.64 | 1.89 (1.42, 2.51)* | 0.64 | 1.89 (1.42, 2.51)* | 0.64 | 1.89 (1.42, 2.51)* | 0.64 | 1.89 (1.42, 2.51)* |
| Q3 | 1.09 | 2.98 (2.29, 3.88)* | 1.09 | 2.98 (2.29, 3.88)* | 1.09 | 2.98 (2.29, 3.88)* | 1.09 | 2.98 (2.29, 3.88)* | 1.09 | 2.98 (2.29, 3.88)* | 1.09 | 2.98 (2.29, 3.88)* |
| Q4 | 1.52 | 4.55 (3.56, 5.83)* | 1.52 | 4.55 (3.56, 5.83)* | 1.52 | 4.55 (3.56, 5.83)* | 1.52 | 4.55 (3.56, 5.83)* | 1.52 | 4.55 (3.56, 5.83)* | 1.52 | 4.55 (3.56, 5.83)* |
| Q5 | 2.00 | 7.37 (5.80, 9.36)* | 2.00 | 7.37 (5.80, 9.36)* | 2.00 | 7.37 (5.80, 9.36)* | 2.00 | 7.37 (5.80, 9.36)* | 2.00 | 7.37 (5.80, 9.36)* | 2.00 | 7.37 (5.80, 9.36)* |
| *P* for trend |  | <0.001 |  | <0.001 |  | <0.001 |  | <0.001 |  | <0.001 |  | <0.001 |
| Model I |  |  |  |  |  |  |  |  |  |  |  |  |
| ALT/AST ratio | 1.51 | 4.53 (3.98, 5.17)* | 1.51 | 4.53 (3.98, 5.17)* | 1.51 | 4.53 (3.98, 5.17)* | 1.51 | 4.53 (3.98, 5.17)* | 1.51 | 4.53 (3.98, 5.17)* | 1.51 | 4.53 (3.98, 5.17)* |
| ALT/AST ratio (Quintile) | | | | | | | | | | | | |
| Q1 | Ref | Ref | Ref | Ref | Ref | Ref | Ref | Ref | Ref | v | Ref | Ref |
| Q2 | 0.64 | 1.90 (1.43, 2.52)* | 0.64 | 1.90 (1.43, 2.52)* | 0.64 | 1.90 (1.43, 2.52)* | 0.64 | 1.90 (1.43, 2.52)* | 0.64 | 1.90 (1.43, 2.52)* | 0.64 | 1.90 (1.43, 2.52)* |
| Q3 | 1.11 | 3.03 (2.32, 3.95)* | 1.11 | 3.03 (2.32, 3.95)* | 1.11 | 3.03 (2.32, 3.95)* | 1.11 | 3.03 (2.32, 3.95)* | 1.11 | 3.03 (2.32, 3.95)* | 1.11 | 3.03 (2.32, 3.95)* |
| Q4 | 1.55 | 4.69 (3.67, 6.01)* | 1.55 | 4.69 (3.67, 6.01)* | 1.55 | 4.69 (3.67, 6.01)* | 1.55 | 4.69 (3.67, 6.01)* | 1.55 | 4.69 (3.67, 6.01)* | 1.55 | 4.69 (3.67, 6.01)* |
| Q5 | 2.03 | 7.62 (6.00, 9.69)* | 2.03 | 7.62 (6.00, 9.69)* | 2.03 | 7.62 (6.00, 9.69)* | 2.03 | 7.62 (6.00, 9.69)* | 2.03 | 7.62 (6.00, 9.69)* | 2.03 | 7.62 (6.00, 9.69)* |
| *P* for trend |  | <0.001 |  | <0.001 |  | <0.001 |  | <0.001 |  | <0.001 |  | <0.001 |
| Model II |  |  |  |  |  |  |  |  |  |  |  |  |
| ALT/AST ratio | 0.74 | 2.09 (1.72, 2.54)* | 0.84 | 2.33 (2.00, 2.70)* | 0.82 | 2.26 (1.95, 2.63)* | 0.87 | 2.40 (2.07, 2.78)* | 0.85 | 2.33 (2.00, 2.71)* | 0.81 | 2.24 (1.93, 2.60)* |
| ALT/AST ratio (Quintile) | | | | | | | | | | | | |
| Q1 | Ref | Ref | Ref | Ref | Ref | Ref | Ref | Ref | Ref | Ref | Ref | Ref |
| Q2 | 0.31 | 1.36 (0.98, 1.89) | 0.47 | 1.60 (1.20, 2.12)* | 0.48 | 1.62 (1.22, 2.15)* | 0.47 | 1.60 (1.21, 2.13)* | 0.44 | 1.55 (1.17, 2.07)* | 0.45 | 1.57 (1.18, 2.09)* |
| Q3 | 0.67 | 1.96 (1.44, 2.68)* | 0.86 | 2.37 (1.81, 3.09)* | 0.87 | 2.38 (1.82, 3.10)* | 0.88 | 2.41 (1.85, 3.15)* | 0.82 | 2.27 (1.74, 2.96)* | 0.82 | 2.28 (1.75, 2.97)* |
| Q4 | 0.92 | 2.50 (1.87, 3.36)* | 1.08 | 2.94 (2.29, 3.78)* | 1.07 | 2.91 (2.27, 3.74)* | 1.08 | 2.93 (2.28, 3.77)* | 1.05 | 2.85 (2.22, 3.66)* | 1.04 | 2.84 (2.21, 3.65)* |
| Q5 | 1.11 | 3.04 (2.27, 4.07)* | 1.31 | 3.70 (2.90, 4.72)* | 1.30 | 3.68 (2.88, 4.70)* | 1.33 | 3.79 (2.97, 4.84)* | 1.29 | 3.62 (2.83, 4.62)* | 1.28 | 3.59 (2.81, 4.58)* |
| *P* for trend |  | <0.001 |  | <0.001 |  | <0.001 |  | <0.001 |  | <0.001 |  | <0.001 |
| Model III |  |  |  |  |  |  |  |  |  |  |  |  |
| ALT/AST ratio | 0.70 | 2.02 (1.61, 2.54)* | 0.84 | 2.31 (1.98, 2.70)* | 0.79 | 2.20 (1.89, 2.57)* | 0.83 | 2.30 (1.97, 2.68)* | 0.83 | 2.28 (1.95, 2.67)* | 0.77 | 2.17 (1.85, 2.54)* |
| ALT/AST ratio (Quintile) | | | | | | | | | | | | |
| Q1 | Ref | Ref | Ref | Ref | Ref | Ref | Ref | Ref | Ref | Ref | Ref | Ref |
| Q2 | 0.27 | 1.32 (0.93, 1.86) | 0.43 | 1.54 (1.16, 2.05)* | 0.46 | 1.58 (1.19, 2.10)* | 0.44 | 1.56 (1.17, 2.07)* | 0.41 | 1.51 (1.14, 2.01)* | 0.40 | 1.49 (1.12, 1.98)* |
| Q3 | 0.48 | 1.61 (1.15, 2.25)* | 0.76 | 2.15 (1.64, 2.81)* | 0.80 | 2.22 (1.70, 2.91)* | 0.79 | 2.20 (1.68, 2.89)* | 0.76 | 2.13 (1.63, 2.79)* | 0.73 | 2.07 (1.58, 2.71)* |
| Q4 | 0.78 | 2.18 (1.59, 2.99)* | 1.02 | 2.77 (2.15, 3.56)* | 1.03 | 2.79 (2.17, 3.59)* | 1.02 | 2.77 (2.15, 3.57)* | 1.01 | 2.74 (2.13, 3.52)* | 0.97 | 2.64 (2.05, 3.40)* |
| Q5 | 0.94 | 2.56 (1.87, 3.52)* | 1.23 | 3.43 (2.68, 4.40)* | 1.24 | 3.45 (2.69, 4.42)* | 1.26 | 3.53 (2.75, 4.53)* | 1.22 | 3.39 (2.64, 4.35)* | 1.19 | 3.29 (2.56, 4.23)* |
| *P* for trend |  | <0.001 |  | <0.001 |  | <0.001 |  | <0.001 |  | <0.001 |  | <0.001 |
| Model IV |  |  |  |  |  |  |  |  |  |  |  |  |
| ALT/AST ratio | 0.71 | 2.02 (1.60, 2.56)* | 0.85 | 2.33 (1.99, 2.73)* | 0.80 | 2.22 (1.89, 2.59)* | 0.83 | 2.28 (1.95, 2.67)* | 0.83 | 2.30 (1.96, 2.70)* | 0.78 | 2.17 (1.85, 2.55)* |
| ALT/AST ratio (Quintile) | | | | | | | | | | | | |
| Q1 | Ref | Ref | Ref | Ref | Ref | Ref | Ref | Ref | Ref | Ref | Ref | Ref |
| Q2 | 0.29 | 1.34 (0.95, 1.89) | 0.43 | 1.54 (1.16, 2.04)* | 0.45 | 1.57 (1.18, 2.09)* | 0.44 | 1.55 (1.16, 2.06)* | 0.41 | 1.51 (1.14, 2.01)* | 0.40 | 1.49 (1.12, 1.98)* |
| Q3 | 0.48 | 1.62 (1.16, 2.26)* | 0.75 | 2.12 (1.62, 2.78)* | 0.79 | 2.20 (1.68, 2.87)* | 0.78 | 2.17 (1.66, 2.84)* | 0.75 | 2.12 (1.62, 2.77)* | 0.72 | 2.06 (1.57, 2.69)* |
| Q4 | 0.78 | 2.17 (1.59, 2.98)* | 1.01 | 2.75 (2.13, 3.54)* | 1.02 | 2.77 (2.15, 3.57)* | 1.00 | 2.73 (2.12, 3.51)* | 1.00 | 2.72 (2.11, 3.50)* | 0.97 | 2.63 (2.04, 3.38)* |
| Q5 | 0.93 | 2.53 (1.84, 3.49)* | 1.23 | 3.43 (2.67, 4.40)* | 1.23 | 3.44 (2.68, 4.41)* | 1.25 | 3.48 (2.71, 4.46)* | 1.22 | 3.39 (2.64, 4.36)* | 1.19 | 3.28 (2.55, 4.21)* |
| *P* for trend |  | <0.001 |  | <0.001 |  | <0.001 |  | <0.001 |  | <0.001 |  | <0.001 |

**P*<0.05; Abbreviations: CI: confidence interval, aHRs: adjusted hazard ratios; other abbreviations as in Table ​2; Crude model adjusted for none; Model I adjusted for age; Model II adjusted for age, GGT, DBIL, TG, HDL-C and BMI; Model III adjusted for age, ALP, GGT, GLB, TB, DBIL, BUN, CR, UA, FPG, TG, HDL-C, Height, BMI, SBP and DBP; Model IV adjusted for age, GGT, ALB, GLB, TB, DBIL, SBP, DBP, ALP, BUN, CR, UA, FPG, TG, HDL-C, LDL-C, Height and BMI.

Supplementary Table 6: Subgroup analysis by stratified cox regression model.

|  | original data | | Post-imputation1 | | Post-imputation2 | | Post-imputation3 | | Post-imputation4 | | Post-imputation5 | |  | |  |
| --- | --- | --- | --- | --- | --- | --- | --- | --- | --- | --- | --- | --- | --- | --- | --- |
|  | β | **aHRs (95%CI)** | β | **aHRs (95%CI)** | β | **aHRs (95%CI)** | β | **aHRs (95%CI)** | β | **aHRs (95%CI)** | β | **aHRs (95%CI)** |  | |  |
| Age (years) | | | | | | | | | | | | | | | |
| ≥70 | 0.42 | 1.53 (0.91, 2.56) | 0.65 | 1.92 (1.23, 3.01)* | 0.69 | 2.00 (1.28, 3.13)* | 0.58 | 1.78 (1.12, 2.82)* | 0.64 | 1.90 (1.21, 2.99)* | 0.65 | 1.92 (1.23, 2.97)* |  | |  |
| ≥60, <70 | 0.87 | 2.39 (1.37, 4.16)* | 0.88 | 2.41 (1.53, 3.80)* | 0.93 | 2.53 (1.61, 3.98)* | 0.93 | 2.54 (1.62, 3.99)* | 0.99 | 2.70 (1.72, 4.24)* | 0.88 | 2.41 (1.54, 3.77)* |  | |  |
| ≥50, <60 | 1.10 | 3.01 (1.98, 4.59)* | 0.90 | 2.45 (1.78, 3.36)* | 0.82 | 2.26 (1.64, 3.12)* | 0.86 | 2.36 (1.72, 3.25)* | 0.92 | 2.50 (1.81, 3.46)* | 0.85 | 2.35 (1.70, 3.23)* |  | |  |
| ≥40, <50 | 0.73 | 2.07 (1.46, 2.93)* | 0.75 | 2.12 (1.64, 2.75)* | 0.74 | 2.10 (1.63, 2.70)* | 0.79 | 2.20 (1.71, 2.82)* | 0.82 | 2.26 (1.75, 2.93)* | 0.79 | 2.21 (1.72, 2.85)* |  | |  |
| ≥30, <40 | 0.67 | 1.96 (1.37, 2.80)* | 0.61 | 1.84 (1.58, 2.16)* | 0.65 | 1.92 (1.63, 2.26)* | 0.59 | 1.81 (1.55, 2.11)* | 0.59 | 1.81 (1.56, 2.10)* | 0.66 | 1.93 (1.63, 2.28)* |  | |  |
| <30 | 0.42 | 1.52 (0.93, 2.48) | 1.01 | 2.77 (2.05, 3.73)* | 1.01 | 2.74 (2.02, 3.73)* | 0.98 | 2.67 (1.97, 3.61)* | 0.98 | 2.67 (1.96, 3.64)* | 0.98 | 2.66 (1.97, 3.59)* |  | |  |
| Sex |  |  |  |  |  |  |  |  |  |  |  |  |  | |  |
| Female | 0.70 | 2.01 (1.60, 2.54)* | 0.67 | 1.96 (1.72, 2.23)* | 0.74 | 2.10 (1.84, 2.40)* | 0.66 | 1.93 (1.71, 2.18)* | 0.66 | 1.94 (1.72, 2.19)* | 0.74 | 2.10 (1.84, 2.39)* |  | |  |
| Male | 0.71 | 2.02 (1.60, 2.56)* | 0.85 | 2.33 (1.99, 2.73)* | 0.80 | 2.22 (1.89, 2.59)* | 0.83 | 2.28 (1.95, 2.67)* | 0.83 | 2.30 (1.96, 2.70)* | 0.78 | 2.17 (1.85, 2.55)* |  | |  |
| SBP, mmHg | | | | | | | | | | | | | | | |
| ≥140 | 0.91 | 2.48 (1.82, 3.38)* | 0.97 | 2.63 (2.03, 3.40)* | 0.91 | 2.49 (1.92, 3.21)* | 0.92 | 2.50 (1.93, 3.23)* | 0.97 | 2.64 (2.04, 3.42)* | 0.95 | 2.59 (2.01, 3.35)* |  | |  |
| <140 | 0.72 | 2.06 (1.69, 2.50)* | 0.70 | 2.02 (1.82, 2.24)* | 0.73 | 2.07 (1.86, 2.31)* | 0.68 | 1.98 (1.79, 2.19)* | 0.69 | 1.99 (1.80, 2.20)* | 0.73 | 2.07 (1.85, 2.31)* |  | |  |
| DBP, mmHg | | | | | | | | | | | | | | | |
| ≥90 | 0.82 | 2.28 (1.54, 3.37)* | 0.88 | 2.42 (1.78, 3.29)* | 0.94 | 2.57 (1.90, 3.49)* | 0.97 | 2.63 (1.94, 3.57)* | 0.91 | 2.48 (1.82, 3.39)* | 0.88 | 2.41 (1.77, 3.27)* |  | |  |
| <90 | 0.72 | 2.06 (1.72, 2.48)* | 0.71 | 2.04 (1.84, 2.25)* | 0.73 | 2.07 (1.86, 2.30)* | 0.67 | 1.96 (1.78, 2.16)* | 0.69 | 2.00 (1.82, 2.20)* | 0.74 | 2.10 (1.89, 2.33)* |  | |  |
| GGT, U/L | | | | | | | | | | | | | | | |
| ≥40 | 0.47 | 1.60 (1.24, 2.06)* | 0.50 | 1.65 (1.37, 1.98)* | 0.48 | 1.62 (1.35, 1.95)* | 0.51 | 1.66 (1.38, 1.99)* | 0.52 | 1.68 (1.40, 2.03)* | 0.51 | 1.66 (1.38, 1.99)* |  | |  |
| <40 | 1.08 | 2.94 (2.36, 3.67)* | 0.80 | 2.23 (2.01, 2.48)* | 0.87 | 2.39 (2.14, 2.67)* | 0.78 | 2.18 (1.97, 2.41)* | 0.79 | 2.21 (2.00, 2.45)* | 0.87 | 2.38 (2.13, 2.65)* |  | |  |
| FPG, mmol/L | | | | | | | | | | | | | | | |
| <3.9 | NA | NA | NA | NA | 0.48 | inf. (0.00, Inf) | 0.01 | inf. (0.00, Inf) | 0.23 | inf. (0.00, Inf) | NA | NA |  | |  |
| ≥3.9,<6.1 | 0.76 | 2.15 (1.79, 2.57)* | 0.72 | 2.05 (1.86, 2.27)* | 0.75 | 2.12 (1.91, 2.35)* | 0.71 | 2.03 (1.84, 2.23)* | 0.71 | 2.03 (1.84, 2.23)* | 0.75 | 2.12 (1.91, 2.36)* |  | |  |
| ≥6.1 | 0.63 | 1.87 (1.22, 2.88)* | 1.02 | 2.77 (1.93, 3.98)* | 0.84 | 2.31 (1.61, 3.30)* | 0.86 | 2.37 (1.66, 3.40)* | 1.00 | 2.73 (1.90, 3.92* | 0.88 | 2.42 (1.68, 3.48)* |  | |  |
| TG, mmol/L | | | | | | | | | | | | | |  |  |
| ≥1.7 | 0.63 | 1.88 (1.49, 2.37)* | 0.80 | 2.22 (1.87, 2.63)* | 0.77 | 2.17 (1.83, 2.57)* | 0.83 | 2.28 (1.93, 2.70)* | 0.81 | 2.25 (1.90, 2.68)* | 0.80 | 2.22 (1.87, 2.63)* |  | |  |
| <1.7 | 0.89 | 2.43 (1.94, 3.05)* | 0.72 | 2.05 (1.82, 2.32)* | 0.77 | 2.14 (1.88, 2.43)* | 0.68 | 1.98 (1.75, 2.23)* | 0.71 | 2.03 (1.80, 2.29)* | 0.76 | 2.13 (1.88, 2.43)* |  | |  |
| TC, mmol/L | | | | | | | | | | | | | |  |  |
| >5.2 | 0.81 | 2.24 (1.62, 3.11)* | 0.88 | 2.42 (1.89, 3.09)* | 0.92 | 2.51 (1.97, 3.21)* | 0.87 | 2.38 (1.86, 3.04)* | 0.92 | 2.52 (1.96, 3.23)* | 0.88 | 2.41 (1.88, 3.09)* |  | |  |
| ≤5.2 | 0.70 | 2.01 (1.67, 2.43)* | 0.72 | 2.05 (1.84, 2.28)* | 0.73 | 2.08 (1.86, 2.33)* | 0.70 | 2.01 (1.81, 2.22)* | 0.70 | 2.01 (1.81, 2.23)* | 0.75 | 2.11 (1.89, 2.36)* |  | |  |

**P*<0.05; Abbreviations: CI: confidence interval, aHRs: adjusted hazard ratios; other abbreviations as in Table ​2; The above model adjusted for sex, age, GGT, ALB, GLB, TB, DBIL, SBP, DBP, ALP, BUN, CR, UA, FPG, TG, HDL-C, LDL-C, Height and BMI.

Supplementary Table 7: nonlinearity among original data and post-imputation data.

|  | Original data | | Post-imputation1 | | Post-imputation2 | | Post-imputation3 | | Post-imputation 4 | | Post-imputation 5 | |
| --- | --- | --- | --- | --- | --- | --- | --- | --- | --- | --- | --- | --- |
|  | β | **aHRs (95%CI)** | β | **aHRs (95%CI)** | β | **aHRs (95%CI)** | β | **aHRs (95%CI)** | β | **aHRs (95%CI)** | β | **aHRs (95%CI)** |
| *Total* |  |  |  |  |  |  |  |  |  |  |  |  |
| Fitting model by standard linear regression | | | | | | | | | | | | |
|  | 0.74 | 2.09 (1.78, 2.46)* | 0.74 | 2.10 (1.91, 2.30)* | 0.76 | 2.15 (1.95, 2.37)* | 0.72 | 2.06 (1.88, 2.25)* | 0.73 | 2.07 (1.89, 2.26)* | 0.77 | 2.16 (1.95, 2.38)* |
| Fitting model by two-piecewise linear regression | | | | | | | | | | | | |
| The inflection point of ALT/AST ratio |  | 0.93 |  | 0.93 |  | 0.93 |  | 0.93 |  | 0.93 |  | 0.93 |
| ≤ 0.93 | 2.67 | 14.41 (8.91, 23.30)* | 2.61 | 13.58 (9.35, 19.71)* | 2.61 | 13.54 (9.34, 19.63)* | 2.60 | 13.52 (9.34, 19.57)* | 2.64 | 14.01 (9.66, 20.32) * | 2.58 | 13.19 (9.08, 19.16) * |
| > 0.93 | 0.01 | 1.01 (0.78, 1.30)* | 0.24 | 1.27 (1.06, 1.50) * | 0.23 | 1.26 (1.06, 1.50) * | 0.24 | 1.27 (1.07, 1.50)* | 0.24 | 1.27 (1.07, 1.50)* | 0.25 | 1.29 (1.08, 1.53)* |
| *P* for log likelihood ratio test |  | <0.001 |  | <0.001 |  | <0.001 |  | <0.001 |  | <0.001 |  | <0.001 |
|  |  |  |  |  |  |  |  |  |  |  |  |  |
| *Male* |  |  |  |  |  |  |  |  |  |  |  |  |
| Fitting model by standard linear regression | | | | | | | | | | | | |
|  | 0.71 | 2.02 (1.60, 2.56)* | 0.80 | 2.23 (2.07, 2.40)* | 0.82 | 2.26 (2.10, 2.44)* | 0.81 | 2.24 (2.08, 2.41)* | 0.80 | 2.24 (2.08, 2.41)* | 0.82 | 2.27 (2.10, 2.44)* |
| Fitting model by two-piecewise linear regression | | | | | | | | | | | | |
| The inflection point of ALT/AST ratio (K) |  | 1.22 |  | 1.16 |  | 1.16 |  | 1.16 |  | 1.16 |  | 1.16 |
| ≤ K | 1.48 | 4.39 (2.97, 6.49)* | 1.82 | 6.19 (5.34, 7.17)* | 1.82 | 6.18 (5.34, 7.16)* | 1.81 | 6.12 (5.28, 7.09)* | 1.82 | 6.17 (5.32, 7.15)* | 1.83 | 6.20 (5.36, 7.19)* |
| > K | -0.42 | 0.65 (0.37, 1.15) | -0.27 | 0.76 (0.64, 0.90)* | -0.24 | 0.79 (0.66, 0.93)* | -0.26 | 0.77 (0.65, 0.91)* | -0.26 | 0.77 (0.65, 0.91)* | -0.24 | 0.79 (0.66, 0.93)* |
| *P* for log likelihood ratio test |  | <0.001 |  | <0.001 |  | <0.001 |  | <0.001 |  | <0.001 |  | <0.001 |
| *Female* |  |  |  |  |  |  |  |  |  |  |  |  |
| Fitting model by standard linear regression | | | | | | | | | | | | |
|  | 0.70 | 2.01 (1.60, 2.54)* | 0.70 | 2.01 (1.89, 2.13)* | 0.68 | 1.98 (1.86, 2.10)* | 0.70 | 2.02 (1.90, 2.14)* | 0.70 | 2.02 (1.90, 2.15)* | 0.68 | 1.97 (1.86, 2.10* |
| Fitting model by two-piecewise linear regression | | | | | | | | | | | | |
| The inflection point of ALT/AST ratio(K) |  | 0.89 |  | 0.91 |  | 0.91 |  | 0.91 |  | 0.91 |  | 0.91 |
| ≤ K | 3.47 | 32.01 (14.18, 72.25)* | 2.87 | 17.57 (13.30, 23.21)* | 2.88 | 17.76 (13.43, 23.47)* | 2.88 | 17.84 (13.50, 23.59)* | 2.85 | 17.28 (13.08, 22.84)* | 2.88 | 17.84 (13.49, 23.58)* |
| > K | -0.19 | 0.83 (0.57, 1.21) | 0.25 | 1.28 (1.15, 1.44)* | 0.23 | 1.26 (1.12, 1.41)* | 0.24 | 1.27 (1.13, 1.42)* | 0.24 | 1.28 (1.14, 1.43)* | 0.22 | 1.25 (1.11, 1.40)* |
| *P* for log likelihood ratio test |  | <0.001 |  | <0.001 |  | <0.001 |  | <0.001 |  | <0.001 |  | <0.001 |

**P*<0.05; Abbreviations: CI: confidence interval, aHRs: adjusted hazard ratios; other abbreviations as in Table ​2; The above model adjust for sex, age, GGT, ALB, GLB, TB, DBIL, SBP, DBP, ALP, BUN, CR, UA, FPG, TG, HDL-C, LDL-C, Height and BMI.
